# Breast imaging at Chris Hani Baragwanath Academic Hospital: A clinically relevant audit

**DOI:** 10.4102/sajr.v24i1.1921

**Published:** 2020-10-15

**Authors:** Ilonka Warnich, Ilana M. Viljoen, Marianne Kuehnast

**Affiliations:** 1 Department of Radiology, Faculty of Health Sciences, University of the Witwatersrand, Johannesburg, South Africa; 2 Baragwanath Academic Hospital, Faculty of Health Sciences, University of the Witwatersrand, Johannesburg, South Africa

**Keywords:** breast cancer, mammography, BI-RADS, breast-imaging audit, diagnostic mammography

## Abstract

**Background:**

Breast cancer is a major cause of morbidity and mortality worldwide. From experience, we have found that the disease burden at Chris Hani Baragwanath Academic Hospital (CHBAH) is unique with an advanced stage at presentation.

**Objective:**

To perform a breast-imaging audit at CHBAH, focused on interpretive performance and disease burden.

**Method:**

Demographic and imaging data were retrospectively collected over a 6-month period. Data collected and derived followed the audit-definitions and rules described within the American College of Radiology–breast-imaging reporting and data system (ACR–BI-RADS) atlas (5th edn.). A comparison was made to benchmark values published by the Radiological Society of North America (RSNA).

**Results:**

A total of 1549 mammography examinations were analysed. The screening subgroup (*n* = 808) revealed 11 cancers with a cancer detection rate (CDR) of 13.6 per 1000 studies and a recall rate of 5.94. The diagnostic subgroup (*n* = 741) revealed 130 cancers with a CDR of 175.4 and an abnormal interpretation rate (AIR) of 39 per 100 studies. Along with the positive predictive values, these performance measures for diagnostic mammography were significantly larger than the RSNA-benchmarks (*p* < 0.0001). In addition, the cancer characteristics showed a greater histological mean tumour length, a lower percentage of minimal cancers (defined as ductal carcinoma *in situ* [DCIS] and invasive cancers ≤ 1 cm) and fewer nodal-negative cancers (*p* < 0.0001), in keeping with a more advanced loco-regional stage at presentation.

**Conclusion:**

The study illustrates the challenges faced by a South African breast-imaging unit confronted with advanced loco-regional disease. The cancer burden is highlighted within a community where there is a lack of national screening mammography. The process of performing a basic, clinically relevant audit is simple and should be a routine practice in breast-imaging units.

## Introduction

Breast cancer is a major cause of morbidity and mortality worldwide. It is the most common type of female cancer and the leading cause of cancer deaths amongst women.^1^

The need for standardisation in breast imaging has led to the development of the breast-imaging reporting and data system (BI-RADS) by the American College of Radiology (ACR), as summarised in Table 1.^2^ Breast-imaging findings are categorised according to the suspicion of malignancy.

**TABLE 1:** American College of Radiology breast-imaging reporting and data system final assessment categories.

Category	Management	Probability of cancer
0. Need additional imaging or prior examinations	Recall for additional imaging and/or await prior examination(s)	N/A
1. Negative	Routine screening	Essentially 0%
2. Benign	Routine screening	Essentially 0%
3. Probably benign	Short interval follow-up or continued surveillance mammography	> 0%, but ≤ 2%
4. Suspicious of malignancy	Tissue diagnosis	> 2%, but < 95%
4a. Low suspicion		a. > 2%, but ≤ 10%
4b. Moderate suspicion		b. > 10%, but ≤ 50%
4c. High suspicion		c. > 50%, but < 95%
5. Highly suggestive of malignancy	Tissue diagnosis	≥ 95%
6. Known biopsy-proven malignancy	Surgical excision when clinically appropriate	N/A

*Source:* Sickles EA, D’Orsi CJ. ACR BI-RADS® Follow-up and outcome monitoring. In: ACR BI-RADS® Atlas, Breast imaging reporting and data system. Reston, VA: American College of Radiology, 2013; p. 21–31.

N/A, not applicable.

Regardless of standardised reporting systems, there still exists an inter-user variation in the interpretive performance of breast imaging and the threshold to obtain tissue diagnosis. Audits have an essential role in monitoring performance within a facility.^3^

The ACR outlines *the basic clinically relevant audit* in the ‘follow-up and outcome monitoring’ chapter within the ACR–BI-RADS atlas (5th edn.).^4^ Annual audits are recommended. The relevance of the audit will be directly proportional to the number of metrics evaluated and should, therefore, be as comprehensive as possible. Separate audits on screening and diagnostic studies are advised, as these show significant statistical differences.^4^

Audit guidelines and definitions are provided within the BI-RADS manual (Table 2). Three scenarios for a positive mammogram are described^4^:

- A screening mammogram leading to anything other than routine follow-up (BI-RADS categories 0, 3, 4 and 5).
- A study leading to the recommendation for tissue diagnosis (BI-RADS 4 and 5).
- A study leading to tissue diagnosis being obtained (BI-RADS 4 and 5).

**TABLE 2:** American College of Radiology breast-imaging reporting and data system audit definitions.

Derived data	Definition
True positives (TP)	Positive imaging study with a positive tissue diagnosis of breast cancer.†
False positives (FP)	Positive imaging study with a negative tissue diagnosis for breast cancer.‡
Positive predictive value (PPV)	Reflects true positive cases as a proportion of total positive imaging studies (TP + FP):
1. PPV_1_	1. Based on positive screening cases, with any result other than routine follow-up (BI-RADS categories 0, 3, 4 and 5).
2. PPV_2_	2. Based on positive examinations with the recommendation for tissue diagnosis (BI-RADS 4 and 5).
3. PPV_3_	3. Based on positive examinations where tissue diagnosis was obtained (BI-RADS 4 and 5).
Cancer detection rate (CDR)	Breast cancer-positive cases per 1000 examinations.
Percentage nodal-negative invasive cancers	Reflected as a percentage of total invasive cancer cases.
Percentage ‘minimal’ cancers	Defined as invasive cancer ≤ 1 cm or ductal carcinoma *in situ* (DCIS). Reflected as a percentage of total cancer cases.
Percentage stage 0 or 1 cancers	Reflected as a percentage of total cancer cases.
Abnormal interpretation rate (AIR)/Recall rate	Positive assessments, leading to additional imaging or biopsy, per 100 examinations:
	1. Diagnostic audit: BI-RADS 3, 4, 5.
	2. Screening audit (recall rate): BI-RADS 0, 3, 4, 5.

*Source:* Sickles EA, D’Orsi CJ. ACR BI-RADS® Follow-up and outcome monitoring. In: ACR BI-RADS® Atlas, Breast imaging reporting and data system. Reston, VA: American College of Radiology, 2013; p. 21–31.

BI-RADS, breast-imaging reporting and data system.

† , Breast cancer diagnosed within 12 months following the examination;

‡ , No breast cancer diagnosed within 12 months following the examination.

True and false positive as well as positive predictive values (PPVs) can be derived for each of these scenarios (PPV_1_, PPV_2_ and PPV_3_, respectively). The PPV gives the probability that a positive examination accurately indicates the presence of the disease. Of the subcategories, the PPV_2_ is the most useful within an imaging facility. It is a valuable indicator of interpretive performance as well as the overall biopsy-threshold within the department. The PPV_3_ reflects clinical practice, and should equal PPV_2_, where biopsies were performed on all cases where tissue diagnosis was recommended.^5^

Other valuable performance measures include cancer detection rate (CDR) and abnormal interpretation rate (AIR). Abnormal interpretation rate is referred to as recall rate in screening mammography.^4^

In order for sensitivity and specificity to be derived, negative examinations need to be correlated with a population-based tumour registry to verify the true absence or presence of disease (true- or false-negatives).^4^ No population-based registry is currently available in South Africa.^6^ The data published within the South African national cancer registry lacks certain details required for an audit, such as differentiating between screening and diagnostic studies.^7^ It is acceptable to exclude false negatives, sensitivity and specificity from audits, where it cannot be reliably derived.^4^

Metrics evaluating tumour characteristics, such as invasive cancer size, lymph node status and cancer stage are encouraged to be included. The percentage of minimal cancers, node-negative cancers and metastatic cancers can then be derived. The ACR–BI-RADS atlas defines minimal cancers as invasive cancers ≤ 10 mm or ductal carcinoma *in situ* (DCIS) of any size.^4^ These metrics give an indication of how early disease is detected, which reflects the major goal of screening and early detection programmes within a country.

The ACR–BI-RADS atlas describes the value of comparing a facility’s audit results with acceptable performance parameters. One such value set recommended, is the Radiological Society of North America (RSNA) national performance benchmarks for digital mammography.^4^ Separate publications for diagnostic and screening studies were released in 2017. The data were collected from the breast cancer surveillance consortium (BCSC) and based on the ACR–BI-RADS 5th edition manual.^3,8^

These benchmarks were not intended for use outside the USA, as they reflect the advanced screening programmes and practices specific to the country.^5^ The ACR–BI-RADS manual further describes the limitation of an audit comparison to benchmark data when performed in facilities with relatively small sample sizes, especially the sample obtained from screening-detected cancers. In such cases internal audits or comparison of the facility’s trend over time becomes more useful.^4^

Nonetheless, in our opinion, it is of value to analyse deviation from these international benchmarks. Results need to be interpreted whilst keeping in mind the vast differences in local practices. An incentive to obtain a national mammography database and benchmarks specific to the South African population and resource-limited setting could be motivated for.

The recent addition of tomosynthesis in screening has assisted radiologists in decreasing recall rates,^8^ and is routinely used as an adjunct to standard digital mammography in many practices. Similarly, the concurrent use of ultrasound during the initial examination, contributing to a combined assessment with mammography, will greatly influence the performance of a unit.^4^ The variable use of these modalities is one of the challenges faced with comparative audits in South Africa, as screening practices are adapted to best suit the population it serves. Immediate reading of screening mammography with the variable addition of ultrasound is a standard practice within many South African breast imaging units. Audit results will differ greatly from facilities where batch reading is performed and patients are subsequently recalled for additional imaging, including tomosynthesis and ultrasound, as commonly done in the USA.^9^

Audit results are also dependent on the screening guidelines within a country. Across the globe there is conflicting data and considerable debate on what these recommendations should entail, particularly in the 40–49 year age group.^10^ The ACR recommends women of average risk for the development of breast cancer to commence annual screening mammography from the age of 40.^11^ The United States Preventative Services Task Force (USPSTF) advises biennial screening mammography within the age group 50–74, with the recommendation that women aged 40–49 can have optional screening after discussion with their healthcare provider.^12^

The South African National Department of Health (NDH) released the Breast Cancer Prevention and Control Policy in 2017,^6^ with the major goal of improving breast cancer awareness, early detection and management within the country. Mammography is recognised as the screening method of choice in developed countries, however, South Africa currently lacks the resources to employ and sustain a national screening programme. It is stated that such a programme should only be introduced if it can be ensured that at least 70% of the target population will benefit from it.^6^ A large percentage of women do not have access to screening mammography, especially those within the rural setting. This contributes to a delay in diagnosis and upstaging of disease.^13^ The NDH recommends clinical breast examination and breast self-examination for early detection of disease. It is, however, recognised that such methods have not yet been proven as efficient screening tools.^6^

The current recommendations by the relevant imaging societies within South Africa are in favour of regular screening mammography. The Radiological Society of South Africa (RSSA) and Breast Imaging Society of South Africa (BISSA) advise annual screening from the age of 40, which is in accordance with the recommendation from the ACR.^10,11^

Despite there being no national organised screening programme within the USA, there is a high prevalence of opportunistic screening being performed with a reported 65% compliance rate (2015).^5,14^ Audit results are expected to differ in countries where a lower frequency of screening is done, particularly when evaluating the size and stage of the screen-detected cancers. Earlier detection of tumours are expected when more screening is performed. In addition, because of the lack of surveillance by an organised screening programme, self-funding and the ever increasing risk of malpractice litigation within the USA, the goal of reducing false positive outcomes is deemed less important. This further limits comparative audits with other countries.^5^

Opportunistic screening mammography is also done within South Africa, however, auditing data are generally not kept or available at most facilities.^10^ Therefore, scant research exists on the rate of screening mammography done within the country. The authors suspect the figures to be significantly less than the USA, particularly within the public sector.

There are many countries that do offer national or provincial organised screening programmes, such as Sweden, the Netherlands, Norway, Spain, the United Kingdom, etc. Auditing data from these countries are expected to differ. Numerous observational studies from these programmes provide direct proof of the benefit of screening mammography.^10,15^

Chris Hani Baragwanath Academic Hospital (CHBAH) is a major South African tertiary referral institution serving an extensive drainage area.^16^ A relatively large proportion of patients receive diagnostic rather than screening mammography, most of which are referred from the CHBAH specialist breast clinic. The exception to this is in the month of October, national breast cancer awareness month,^17^ during which screening is promoted.

The relevance of the breast-imaging audit, focused on interpretive performance and disease burden, is to standardise practices as well as to build a breast-imaging database. The results can be utilised to facilitate quality and skill-improvement methods.

## Research methods and design

A retrospective, descriptive study design was used. The objective was to perform a breast-imaging audit at CHBAH, according to the guidelines outlined in the ACR–BI-RADS 5th edition manual.

The study population consisted of patients who received a mammogram between 01 June and 31 November 2018. Cases were excluded where inadequate information was available to classify it as diagnostic or screening. Screening studies were defined as routine investigations done for asymptomatic patients. Clinical data provided on the radiology report were relied upon to determine these cases. Diagnostic studies included the investigation of breast complaints and the short-term follow-up of previous abnormal assessments. Additional exclusion criteria were BI-RADS six assessments and previous breast augmentation or mastectomy. In patients with a unilateral mastectomy, imaging of the contralateral breast was included.

The standard imaging protocol included digital tomosynthesis mammography (Hologic Selenia Dimensions with AWS 8000, Laurel Bridge Software). Two-dimensional images (craniocaudal and mediolateral-oblique views) were created from breast tomosynthesis using C-view software. The mammography imaging protocol was the same for screening and diagnostic investigations. Breast ultrasound (Aloka – ProSound Alpha 10 system, Version 8 Software) was routinely performed, with the occasional exception of patients presenting with low density breasts (ACR-BI-RADS categories A or B) and unchanged follow-up screening mammography (*n* = 156). When indicated, tissue diagnosis was obtained within the mammography unit. This included ultrasound-guided fine-needle-aspiration, core needle and stereotactic biopsies.

Data were collected from the hospital picture archiving and communication system (PACS – AGFA IMPAX 6.5.1.501). Consultant-approved mammography reports were used to obtain the clinical demographics, indications and relevant imaging findings (breast composition and ACR–BI-RADS final assessments). In cases where each breast was assigned a separate BI-RADS, the highest category was used.

Histology results were tracked for all positive studies (BI-RADS 4, 5), as well as any other tissue sampling. Results were obtained from the National Health Laboratory Service (NHLS), using the NHLS LABTRAK web results viewer. Where available, the post-surgical tumour size and nodal involvement were recorded, as stated on the pathology report. These histopathologically proven data were used in the calculation of cancer characteristics. In the presence of multifocal or multicentric disease, the greatest diameter of the largest tumour focus was used as the tumour size.

Data were captured by the primary researcher using Microsoft Excel and the ACR audit definitions and rules were followed (Table 2). It was similar to the methodology used to obtain the RSNA-benchmarks. Derived data were based on mammography examinations with tomosynthesis and additional ultrasound, when performed. Recall rate was defined as any screening investigation with an assessment of BI-RADS 0, 3, 4 or 5. The false-negative values, sensitivity, specificity and presence of metastatic disease were not included in the audit.

Data were analysed using SAS Version 9.2. Descriptive statistics were calculated: numerical data using means with standard-deviations or medians with interquartile ranges (IQRs) and categorical data using frequencies and percentages. The following analytical statistics were used to compare the sample statistics with the published benchmarks: the single proportion binomial test to compare the sample proportion with a proportion in the published benchmarks; the one-sample *t*-test to compare the sample-mean with a mean in the published benchmarks; the one-sample Wilcoxon-signed-rank test to compare the sample-median with a median in the published benchmarks. The Shapiro–Wilk test was used to investigate if the numerical variables were normally distributed. A significance level of *p* < 0.05 was used.

### Ethical consideration

Ethical approval was obtained from the Human Research Ethics Committee, University of the Witwatersrand. Clearance certificate number: M190458.

## Results

A total of 1549 mammography examinations were included in the audit, consisting of 808 (52.16%) screening and 741 (47.84%) diagnostic studies. Table 3 shows the demographic and breast-imaging data collected and the cancers detected within the various subgroups. The vast majority (79.66%) of the patients had predominantly fatty or scattered fibroglandular density breast composition (ACR-BI-RADS category A or B). The breast-density distribution showed no significant difference amongst the cancer-positive cases, compared with the non-cancer cases (screening: *p* = 0.4114, diagnostic: *p* = 0.0877).

**TABLE 3:** Audit results for 1549 mammography examinations.

Characteristic	Screening				Diagnostic			
	Total number of examinations	%	Number of cancer-positive cases	%	Total number of examinations	%	Number of cancer-positive cases	%
**Age (years)**								
< 30	3	0.37	0		16	2.16	5	3.85
30–39	44	5.45	1	9.09	164	22.13	23	17.69
40–49	207	25.62	4	36.36	214	28.88	19	14.62
50–59	247	30.57	2	18.18	160	21.59	37	28.46
60–69	195	24.13	3	27.27	127	17.14	23	17.69
70–79	86	10.64	1	9.09	48	6.8	16	12.31
≥ 80	26	3.22	0	-	12	1.62	7	5.38
**Gender**								
Female	808	100	11	100	692	93.39	130	100
Male	0	-	0	-	49	6.61	0	-
**Personal history of breast cancer**								
Yes	277	34.28	6	54.55	24	3.24	4	3.08
No	531	65.72	5	45.45	717	96.76	126	96.92
**Breast composition**								
Predominantly fatty (A)	318	39.36	2	18.18	216	29.15	34	26.15
Scattered fibroglandular density (B)	373	46.16	6	54.55	325	43.86	62	47.69
Heterogeneously dense (C)	66	8.17	1	9.09	100	13.50	13	10
Extremely dense (D)	6	0.74	0		19	2.56	5	3.85
Not specified (N/S)	45	5.57	2	18.18	81	10.93	16	12.31
**BI-RADS classification**								
0	0	-	0	-	0	-	0	-
1	295	36.51	N/A	-	123	16.06	N/A	-
2	464	57.43	N/A	-	308	41.57	N/A	-
3	12	1.49	0	-	63	8.50	1	0.77
4 – N/S	1	0.12	0	-	8	1.08	2	1.54
4a	18	2.23	3	27.27	64	8.64	10	7.69
4b	5	0.62	1	9.09	22	2.97	4	3.08
4c	5	0.62	1	9.09	13	1.75	5	3.85
5	7	0.87	6	54.55	119	16.0	105	80.77
Not given	1	0.12	0	-	21	2.83	3	2.31
**Total**	**808**	**-**	**11**	**-**	**741**	**-**	**130**	**-**

BI-RADS, breast-imaging reporting and data system.

Note: Percentages are based on the total examinations within each column (total values are reported in the first row).

N/A, not applicable (the number of cancer-positive cases were not assessed for BI-RADS 1 and 2).

The majority of the final assessments were BI-RADS 1 or 2 (regarded as negative examinations), within both the screening (93.94%) and the diagnostic (58.17%) subgroups.

**FIGURE 1:**
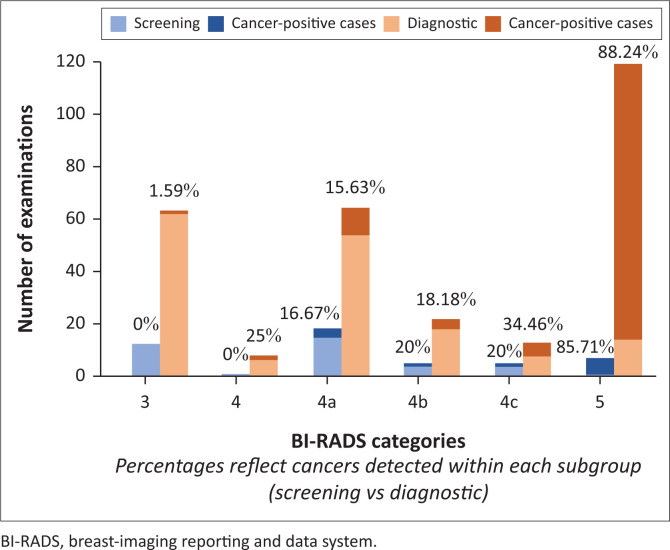
Breast-imaging reporting and data system categories and cancers detected for abnormal interpretations in screening and diagnostic mammography.

Abnormal interpretations included BI-RADS categories 3, 4 and 5. The distribution of cancers detected within each of these subcategories is illustrated in Figure 1. The majority of abnormal interpretations and cancers detected were from the diagnostic studies.

Further analysis was done separately for screening and diagnostic studies.

### Screening

The patients were all female and 94.18% were over the age of 40 years. The median age screened was 56 (interquartile range [IQR] 48.0–64.0), ranging from 23 to 91 years. A known history of previously treated breast cancer was seen in 34.28% (*n* = 277). The median age of cancer-positive cases was 55 (IQR 47.0–62.0), with a minimum of 39 and a maximum of 71 years (Table 3).

The majority of patients underwent the standard mammography imaging protocol with digital breast tomosynthesis and additional ultrasound (*n* = 652, 80.67%). The remaining studies consisted of mammography with tomosynthesis only (*n* = 156, 19.31%), and represented patients who came for follow-up screening mammography with low density breast parenchyma (ACR–BI-RADS categories A or B) and unchanged negative assessments (BI-RADS 1 or 2).

Abnormal interpretations (BI-RADS 0, 3, 4 or 5) were reported in 48 of the 808 screening studies, resulting in a recall rate/AIR of 5.94 (Table 4)^8^. These patients were all evaluated using mammography, tomosynthesis and ultrasound and given a final assessment. No patients were categorised as BI-RADS 0. Positive interpretations (BI-RADS 4 or 5) constituted 36 studies. Amongst these, tissue diagnosis was obtained in 32 cases (88.89%). Eleven cancer cases were diagnosed after a positive screening mammogram, with a CDR of 13.6 per 1000 studies. The PPV_1_, PPV_2_ and PPV_3_ were 22.92, 30.56 and 34.38, respectively.

**TABLE 4:** Derived performance measures for screening mammography (*n* = 808).

Measure	CHBAH audit value	95% CI	RSNA-benchmark value	95% CI	*p*
**Recall rate (per 100 studies)**	5.94	4.51, 7.79	11.6	11.5, 11.6	< 0.0001
Number of abnormal interpretations	48	-	194 668	-	-
Total number of examinations	808	-	1 682 504	-	-
**Cancer detection rate (per 1000 studies)**	13.6	7.6, 24.2	5.1	-	0.0006
Number of cancers detected	11	-	8529	-	-
Total number of examinations	808	-	1 682 504	-	-
**PPV_1_**	22.92	13.31, 36.54	4.4	4.3, 4.5	< 0.0001
Number of cancers detected	11	-	8529	-	-
BI-RADS 0, 3, 4, 5	48	-	194 668	-	-
**PPV_2_**	30.56	18.01, 46.86	25.6	25.1, 26.1	0.4976
Number of cancers detected	11	-	7376	-	-
BI-RADS 4, 5	36	-	28 785	-	-
**PPV_3_**	34.38	20.41, 51.69	28.6	28.0, 29.3	0.4708
Number of cancers detected	11	-	5945	-	-
BI-RADS 4, 5 with biopsy	32	-	20 763	-	-

*Source*: Lehman C, Arao R, Sprague B, et al. National performance benchmarks for modern screening digital mammography: Update from the breast cancer surveillance consortium. Radiology. 2017;283(1):49–58. https://doi.org/10.1148/radiol.2016161174

CI, confidence interval; CHBAH, Chris Hani Baragwanath Academic Hospital; RSNA, Radiological Society of North America; PPV, positive predictive value; BI-RADS, breast-imaging reporting and data system.

There were nine invasive cancers and two DCIS lesions (Table 5)^8^. The invasive cancer cases with a known pathological tumour size (*n* = 5) and nodal status (*n* = 5) demonstrated the following cancer characteristics (Table 5): median tumour length of 20.00 mm (IQR 9.5–26), minimal-cancer rate of 42.9% (*n* = 3) and nodal-negative cancer rate of 60% (*n* = 3). There were no synchronous bilateral, multicentric or multifocal cancers detected.

**TABLE 5:** Derived cancer characteristics for screening mammography (*n* = 11).

Characteristic	CHBAH audit value	%	RSNA-benchmark value	%	*p*
**Cancer type**	-	-	-	-	0.5202
Ductal carcinoma *in situ* (DCIS)†	2	18.2	2644	31.0	-
Low grade	0	-	Unknown	-	-
Intermediate grade	1	-	Unknown	-	-
High grade	1	-	Unknown	-	-
Invasive	9	81.8	5885	69.0	-
**Invasive cancer size (mm)**‡					0.5782
1–5	0	-	727	12.7	-
6–10	1	20	1461	25.6	-
11–15	1	20	1459	25.5	-
16–20	2	40	840	14.7	-
> 20	1	20	1228	21.5	-
Unknown	4	-	170	-	-
**Minimal cancer§**	-	-	-	-	0.4658
Yes	3	42.9	4816	57.7	-
No	4	57.1	3527	42.3	-
Unknown	4	-	186	-	-
**Axillary lymph node status (invasive cancer)¶**	-	-	-	-	0.2745
Positive	2	40.00	1190	20.6	-
Negative	3	60.00	4599	79.4	-
Unknown	4	-	96	-	-
**HIV-status**	-	-	-	-	-
Positive	2	33.33	Unknown	-	-
Negative	4	66.67	Unknown	-	-
Unknown	5	-	Unknown	-	-
**Total number of cancers**	11	-	8529	-	-

*Source*: Lehman C, Arao R, Sprague B, et al. National performance benchmarks for modern screening digital mammography: Update from the breast cancer surveillance consortium. Radiology. 2017;283(1):49–58. https://doi.org/10.1148/radiol.2016161174

CHBAH, Chris Hani Baragwanath Academic Hospital; RSNA, Radiological Society of North America; HIV, human immunodeficiency virus.

† , DCIS post-surgical tumour size as measured on pathology specimen, where available (*n* = 1): intermediate-grade 100 mm; high-grade unknown size; ‡, Invasive cancer post-surgical tumour size as measured on pathology specimen, where available (*n* = 5). Median 20.00 mm (interquartile range 9.5–26), mean 18.2 mm (standard deviation 9.5); benchmark mean 15.9 mm (*p* = 0.3043); §, Defined as ductal carcinoma *in situ* or invasive cancer ≤ 10 mm; ¶, Refers only to invasive cancers with available nodal pathology results (*n* = 5).

The positive cancer cases within the subgroup of women who had a known history of previously treated breast cancer included the following: loco-regional invasive cancer recurrence after previous breast conserving therapy (*n* = 2; known tumour size 20 mm [*n* = 1]); invasive cancer involving the contralateral breast (*n* = 4; known tumour size 12 mm [*n* = 1]). These patients were all asymptomatic and presented for surveillance screening mammography.

The subgroup of patients with no history of previously treated breast cancer (*n* = 5) showed the following cancer characteristics: CDR of 9.42 (95% CI [4, 21.8]), invasive cancer median tumour length of 20 mm (*n* = 3), two DCIS lesions of intermediate- (*n* = 1; 100 mm) and high-grade (*n* = 1; unknown size). The age distribution of screen-detected cancers within this subgroup of women were as follows (years): 40–49 (*n* = 3, 60%), 50–59 (*n* = 2, 40%), median age of 48 (IQR 45–55.5). These index cancer diagnoses were all screened during the months of October (national breast cancer awareness month) and the beginning of November. Three of these patients represented baseline screening studies, whilst the remaining two patients presented for follow-up screening.

### Diagnostic

Similar audit results were derived for the diagnostic subgroup.

The study population consisted of 93.39% (*n* = 693) female and 6.61% (*n* = 49) male patients. The median age was 48 (IQR 40.0-60.0), with a range of 19-91 years. A personal history of breast cancer was recorded in 3.24% (*n* = 24). The median age of cancer-positive cases was 54.5 (IQR 41.0–65.0), with a minimum of 26 and a maximum of 91 years (Table 3).

Amongst the male patients (*n* = 49), five had abnormal interpretations of their mammograms, consisting of BI-RADS 3 (*n* = 2), BI-RADS 4c (*n* = 2) and BI-RADS 5 (*n* = 1) assessments. Two core biopsies were performed, yielding negative results with no male-detected breast cancers.

**FIGURE 2:**
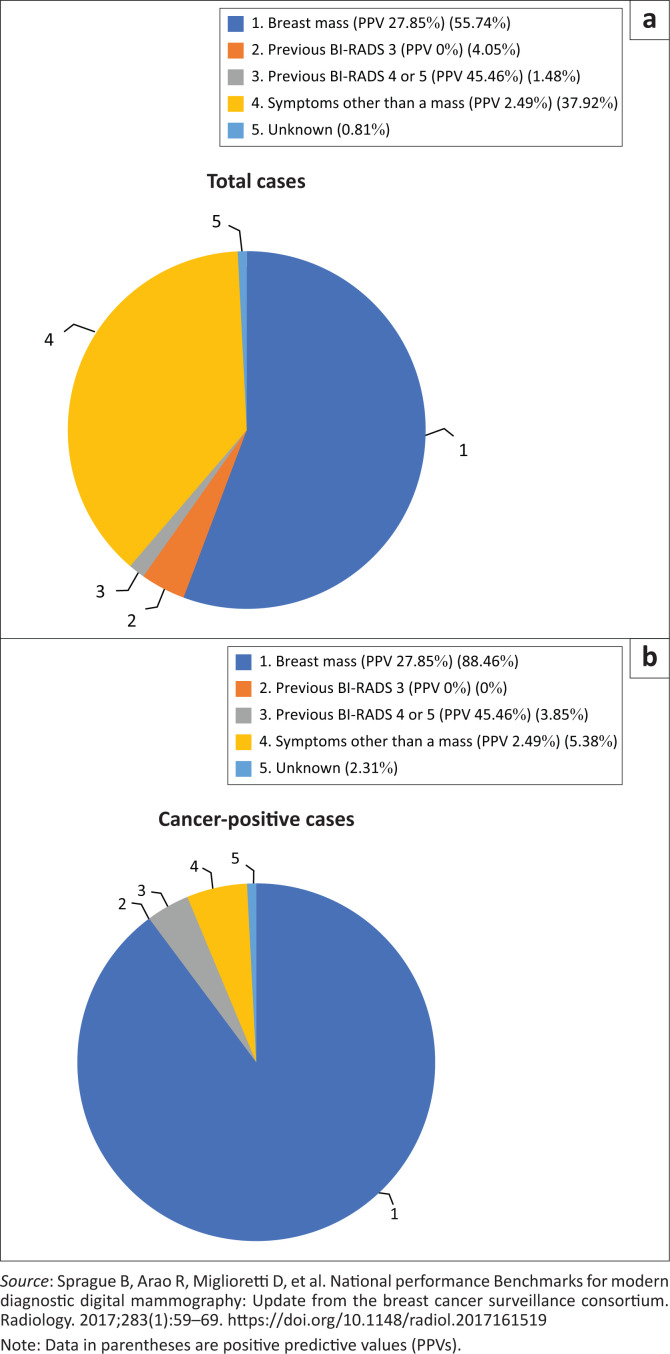
Indications for diagnostic mammography: Total examinations and cancer-positive cases.

The presence of a palpable breast mass constituted 55.74% (*n* = 413) of the diagnostic indications (Figure 2). Amongst these patients presenting with a mass, 27.85% (*n* = 115) of the studies resulted in a diagnosis of cancer, contributing 88.46% to the cancer cases detected within the diagnostic subgroup.

The remainder of the indications included: mastalgia (*n* = 176, PPV = 2.27%), nipple discharge (*n* = 36, PPV = 5.56%), nipple retraction (*n* = 2, PPV = 0%), skin changes (*n* = 9, PPV 11.11%), breast abscess (*n* = 3, PPV = 0%), gynaecomastia (*n* = 31, PPV = 0%), axillary lymph nodes (*n* = 4, PPV = 0%), non-specified breast symptoms (*n* = 17, PPV = 0%), previous BI-RADS 3 (*n* = 30, PPV = 0%) and previous BI-RADS 4 or 5, where no histology was obtained (*n* = 11, PPV = 45.46%).

There were 289 (39%) abnormal interpretations (BI-RADS 3, 4 or 5) and 226 (30.50%) positive studies (BI-RADS 4 or 5). In 98.23% (*n* = 222) of the positive studies, tissue diagnosis was obtained within the unit.

The performance measures (Table 6)^3^ revealed an AIR of 39, CDR of 175.4 per 1000 studies (*n* = 130) and a PPV_2_ and PPV_3_ of 55.75 and 56.76, respectively. These metrics were all significantly higher than the RSNA-benchmark values (*p* < 0.0001).

**TABLE 6:** Derived performance measures for diagnostic mammography (*n* = 741).

Measure	CHBAH audit value	95% CIs	RSNA-benchmark value	95% CI’s	*p*
**Abnormal interpretation rate (per 100 studies)**	39	35.55, 42.56	12.6	12.5, 12.7	< 0.0001
Number of abnormal interpretations	289	-	50 659	-	-
Total number of examinations	741	-	401 548	-	-
**Cancer detection rate (per 1000 studies)**	175.4	149.7, 204.4	34.7	34.1, 35.2	< 0.0001
Number of cancers detected	130	-	13 915	-	-
Total number of examinations	741	-	401 548	-	-
**PPV_2_**	55.75	49.23, 62.08	27.5	27.1, 27.9	< 0.0001
Number of cancers detected	126	-	13 915	-	-
BI-RADS 4, 5	226	-	50 659	-	-
**PPV_3_**	56.76	50.18, 63.32	30.4	29.9, 30.9	< 0.0001
Number of cancers detected	126	-	10 725	-	-
BI-RADS 4, 5 with biopsy	222	-	35 275	-	-

*Source*: Sprague B, Arao R, Miglioretti D, et al. National performance Benchmarks for modern diagnostic digital mammography: Update from the breast cancer surveillance consortium. Radiology. 2017;283(1):59–69. https://doi.org/10.1148/radiol.2017161519

CHBAH, Chris Hani Baragwanath Academic Hospital; RSNA, Radiological Society of North America; CI, confidence interval; PPV, positive predictive value; BI-RADS, breast-imaging reporting and data system.

The vast majority, 96.15% (*n* = 125), of the cancers detected were invasive and the remaining 3.85% (*n* = 5) were DCIS (Table 7). One patient with low-grade DCIS presented with a nipple discharge, whilst the remaining intermediate-grade (*n* = 3) and high-grade (*n* = 1) DCIS lesions were palpable masses. The median tumour size for DCIS lesions on available pathological specimens was 20 mm (*n* = 2). Six patients presented with synchronous bilateral breast cancer (4.62%), six with unilateral multicentric cancer (4.62%) and seven with multifocal cancer (5.39%).

**TABLE 7:** Derived cancer characteristics for diagnostic mammography (*n* = 130).

Characteristic	CHBAH audit value	%	RSNA-benchmark value	%	*p*
**Cancer type**	-	-	-	-	< 0.0001
Ductal carcinoma *in-situ* (DCIS)†	5	3.85	3329	23.9	-
Low-grade	1	-	Unknown	-	-
Intermediate-grade	3	-	Unknown	-	-
High-grade	1	-	Unknown	-	-
Invasive	125	96.15	10 586	76.1	-
**Invasive cancer size (mm)**‡	-	-	-	-	< 0.0001
1–5	3	5.17	955	9.5	-
6–10	2	3.45	1858	18.4	-
11–15	1	1.72	2049	20.3	-
16–20	4	6.9	1444	14.3	-
> 20	48	82.76	3767	37.4	-
Unknown	67	-	513	-	-
**Minimal cancer**§	-	-	-	-	< 0.0001
Yes	10	15.87	6097	45.6	-
No	53	84.13	7260	54.4	-
Unknown	67	-	558	-	-
**Axillary lymph node status (invasive cancer)**¶	-	-	-	-	< 0.0001
Positive	44	69.84	3083	30.4	-
Negative	19	30.16	7074	69.6	-
Unknown	62	-	429	-	-
**HIV-status**	-	-	-	-	-
Positive	34	69.91	Unknown	-	-
Negative	79	30.09	Unknown	-	-
Unknown	17	-	Unknown	-	-
**Total number of cancers**	130	-	13 915	-	-

*Source*: Sprague B, Arao R, Miglioretti D, et al. National performance Benchmarks for modern diagnostic digital mammography: Update from the breast cancer surveillance consortium. Radiology. 2017;283(1):59–69. https://doi.org/10.1148/radiol.2017161519

CHBAH, Chris Hani Baragwanath Academic Hospital; RSNA, Radiological Society of North America; HIV, human immunodeficiency virus.

† , DCIS post-surgical tumour size as measured on pathology specimen, where available (*n* = 2): low-grade unknown size; intermediate-grade 18 mm; high-grade 22 mm; ‡, Invasive cancer post-surgical tumour size as measured on pathology specimen, where available (*n* = 58): median 31 mm (interquartile range 23–45), mean 36.3 mm (standard deviation 23.9); benchmark mean 21.2 mm (*p* < 0.0001); §, Defined as ductal carcinoma *in situ* or invasive cancer ≤ 10 mm; ¶, Refers only to invasive cancers with available nodal pathology results (*n* = 63).

**FIGURE 3:**
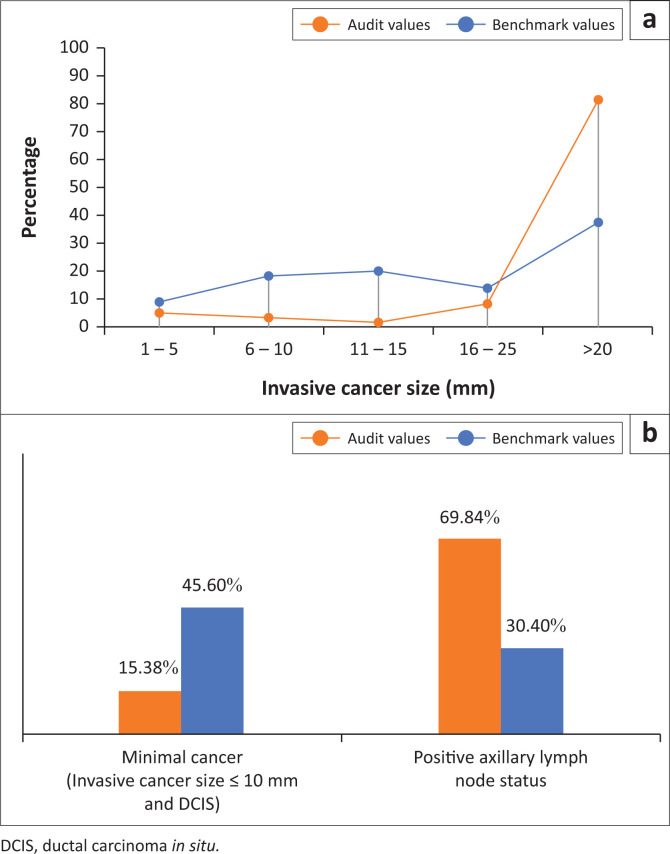
Comparative illustration of diagnostic audit values and benchmark values for cancer characteristics based on known pathological cancer size and axillary lymph node status.

Amongst the invasive cancers with a known pathological tumour size (*n* = 58), 82.76% were greater than 20 mm with a median tumour length of 31 mm (IQR 23.0–45.0). Ten cases (15.87%) were defined as minimal cancers. In approximately half of the cancer-positive cases (50.4%, *n* = 63) the pathological nodal status was available. Of these, 69.84% (*n* = 44) were nodal-positive and 30.16% (*n* = 19) nodal-negative. Figure 3 illustrates the comparison of these tumour characteristics with the RSNA-benchmark values,^3^ depicting a larger mean invasive cancer size with a lower percentage of minimal and nodal-negative cancers (*p* < 0.0001).

## Discussion

As expected, the data derived from the screening and diagnostic subgroups revealed important differences, illustrating the value of performing separate audits.

### Screening

The comparison of the screening audit to RSNA-benchmark values was limited, as expected from the literature review. This was because of the small number of screening-detected cancers limiting the statistical significance of comparing cancer characteristics. Additionally, the differences in screening practices limited the clinical significance of comparing performance measures. The only parameters that showed a statistically significant difference were a lower recall rate/AIR (*p* < 0.0001), a higher PPV_1_ (*p* < 0.0001) and a higher CDR (*p* = 0.0006).

The screening audit included patients with a known history of previous breast cancer (*n* = 277, 34.28%). The ACR audit guidelines advise for these asymptomatic patients to be regarded as screening investigations. Six (54.55%) of the 11 cancers detected were from this high-risk subgroup. In the RSNA screening audit, patients with previous breast cancer constituted only 5.1% of the total patients screened (*n* = 61 628) and 15% of the cancer cases diagnosed (*n* = 1022).^6^ The majority of patients who received screening mammography at CHBAH had previously presented with a breast complaint, including breast cancer. Therefore, the screening population at CHBAH likely represented a high-risk group for the development of breast cancer, impacting the CDR. The exception to this was during the month of October (national breast cancer awareness month), during which screening was promoted. Most of the cancers in the subgroup of women with no history of previous breast cancer were detected during this screening-period, likely representing a more accurate estimate of the CDR in women of average risk within our setting.

The difference in recall rate and PPV_1_ can be explained by the difference in screening practices,and is largely influenced by the number of BI-RADS 0 cases. In screening practices where batch reading of mammography is performed, patients are often assessed as BI-RADS 0 and recalled for additional mammography views, tomosynthesis and/or ultrasound. This is standard practice within most facilities in the USA. At CHBAH, immediate reading of screening mammography with tomosynthesis and additional breast ultrasounds are generally performed at the time of first presentation. This is important within a practice serving a large drainage area, where accessibility to breast-imaging units is poor with difficulties in patient recall and follow-up. Consequently, there were no patients categorised as BI-RADS 0 and the recall rate included final assessments of BI-RADS 3, 4 and 5. This resulted in a significantly lower recall rate and higher PPV_1_.

The relative larger size of screen-detected cancers with fewer minimal and nodal-negative invasive cancers, as compared with the RSNA-benchmarks, could be related to the higher frequency of opportunistic screening being performed within the USA. Moreover, the lower PPVs in the RSNA-benchmarks could be attributed to the specific screening environment within the USA (especially regarding self-funding and litigation), where less emphasis is placed on reducing false positive examinations. Despite the comparison of these audit metrics not being statistically significant, the findings are in keeping with what could be expected from the literature review.

The results should, therefore, be interpreted with caution and reflect the differences in screening practices rather than improved performance. This highlights the limitation of comparative audits to international benchmarks. A comparison with follow-up audits in the same unit or units with similar screening practices would be valuable.

The median age of screen-detected cancers was 55 years, which proved to be younger than what was found in the RSNA screening benchmarks. The 40–49 and 50–59 year age groups collectively contributed the majority of cancers detected (36.36% and 18.18%, respectively), compared with the RSNA-benchmarks where most cancers were detected in women over the age of 60 years (56.3%). In addition, within the subgroup of patients with no personal history of previous breast cancer (*n* = 5), all screen-detected cancers were in the 40–49 and 50–59 year age groups, (60% and 40%, respectively), with a median age of 48 years. This indicates that screening in our setting should be commenced at the age of 40 and would especially benefit women in their 40s and 50s.

The vast majority of patients, in both the screening and diagnostic audits, had low density breasts (type A or B). This could be related to the demographics of the study population, however, would need further investigation.

### Diagnostic

The presence of a palpable breast mass was an important discriminator amongst the reported indications. It was the most common presenting breast complaint and contributed to the majority of cancer cases (88.46%) in the diagnostic subgroup. This was similar to what was reported within the RSNA diagnostic audit. On follow-up audits, the results could be subdivided into ‘mass’ and ‘non-mass’ categories, with a different set of performance measures and tumour characteristics for each group.

The performance measures within the diagnostic audit revealed higher CDR and PPVs, as compared with the RSNA-benchmarks. This may be attributed to the fact that patients presented with a significantly larger mean tumour length. The lower percentages of minimal and nodal-negative cancers further reflect an advanced loco-regional stage at presentation.

The reason for the discrepancy between the number of biopsies advised for positive examinations (from which PPV_2_ is derived) and the number of biopsies performed (from which PPV_3_ is derived) is not clear. Because of the risk of patients defaulting on follow-up, the department strives to perform immediate image-guided biopsies on all positive imaging examinations.

The marked differences in the performance measures as well as tumour characteristics compared with the RSNA-benchmarks are likely linked to factors contributing to late presentation of disease within our setting. This could include poor breast cancer awareness and accessibility to healthcare facilities, including the lack of a national mammography screening programme. Chris Hani Baragwanath Academic Hospital is a referral centre with a large component of diagnostic studies. There are also multiple delays in the successful diagnosis and referral of these patients by the referring hospitals, mostly because of various human and other resource constraints. Another consideration may be a more aggressive nature of disease in our population. Further investigation in this regard is needed.

The results highlight the need for the promotion of breast cancer awareness and education to all South African women. Furthermore, providing mammography screening facilities in local clinics would increase adherence to recommended screening guidelines and greatly improve early detection and downstaging of cancers.

### Study limitations

In addition to the previously mentioned limitations of performing comparative audits using international benchmarks, the following points were noted.

General audit limitations:

1. The lack of availability of a national tumour registry precluded the evaluation of metrics, such as false-negative values, sensitivity and specificity.
2. The unit does not have a routine patient self-questionnaire. Patient referral forms were relied upon for clinical data acquisition and, where inadequate, the study was omitted. Patient questionnaires within the unit could have facilitated more accurate clinical data collection.

Additional limiting factors on comparison of the audit results with the benchmark values:

- The audit was performed over 6 months from studies done in 2018, whereas the benchmark articles included studies from 2007 to 2013.
- At CHBAH, the BI-RADS assessment is based on digital tomosynthesis mammography with the addition of ultrasound in most cases. The RSNA-benchmark data is based on digital mammography alone.
- Male patients were included in our diagnostic audit, whereas the RSNA-benchmarks were limited to female patients only. This, however, only constituted a small number of abnormal interpretations (*n* = 5), with no contribution to the cancers detected.
- Chris Hani Baragwanath Academic Hospital is an academic referral institution, whereas the data collected for the RSNA-benchmarks were largely from non-academic, community-based institutions.

## Recommendations for future research

Regular follow-up audits within a facility would be of great value for continuous quality control, especially when a change in practice is implemented. Performance measures could be obtained for each interpreting radiologist. In addition, comparative audits within different South African breast imaging units would provide valuable comparisons of local practices.

We propose a *modified recall rate* to be used in the auditing of screening practices in South Africa. This should be based on the final BI-RADS assessment after further evaluation with digital breast tomosynthesis and ultrasound is complete, as was done in this study. A final BI-RADS assessment leading to anything other than routine follow-up screening mammography (BI-RADS 3, 4 and 5) should be regarded as an abnormal interpretation and used to calculate the *modified recall rate.* This would provide a metric distinct from the internationally accepted *recall rate*, which would be more applicable to many South African screening practices and allow for more relevant comparisons and future research within the country.

The data obtained from these audits could contribute to a breast-imaging database, providing a baseline for the development of benchmarks and recommendations appropriate to the South African setting.

## Conclusion

The study highlights the unique challenges faced by a breast-imaging unit within a tertiary, South African, government-hospital setting. A large proportion of diagnostic mammography is being performed on a population presenting with advanced loco-regional disease, as compared with international, first world benchmarks. It further illustrates the cancer burden within a community where there is a lack of national screening mammography programmes and the additional need for breast cancer awareness.
